# Development and Validation of Machine Learning-Based Models for Predicting Postoperative Depression Risk in Patients With Ovarian Cancer

**DOI:** 10.62641/aep.v54i2.2177

**Published:** 2026-04-15

**Authors:** Jitong Zhao, Kaige Pei, Junhan Liu, Ce Bian, Chen Ling

**Affiliations:** ^1^Department of Gynecology and Obstetrics, West China Second University Hospital, Sichuan University, 610041 Chengdu, Sichuan, China; ^2^Key Laboratory of Obstetrics and Gynecologic and Pediatric Diseases and Birth Defects of Ministry of Education, West China Second University Hospital, Sichuan University, 610041 Chengdu, Sichuan, China

**Keywords:** ovarian cancer, postoperative depression, machine learning, prediction model, random forest

## Abstract

**Objective:**

To develop machine learning-based prediction models for postoperative depression risk in patients with ovarian cancer and to evaluate their predictive performance and clinical application value.

**Methods:**

Clinical data from 850 postoperative patients with ovarian cancer were retrospectively analysed. Postoperative depression risk was defined as positive when Patient Health Questionnaire-9 (PHQ-9) score was ≥10. Feature selection was performed using least absolute shrinkage and selection operator (LASSO) regression and Boruta algorithm, with the intersection of both methods determining the final predictive variables. Data were randomly divided into training and validation sets at a 7:3 ratio. Five prediction models were constructed: logistic regression, random forest, support vector machine, extreme gradient boosting (XGBoost), and neural network. Model performance was evaluated through area under the receiver operating characteristic curve (AUC), Brier score, calibration curves, and decision curve analysis. SHapley Additive exPlanations (SHAP) method was employed to interpret the feature contributions of the optimal model, and a nomogram was constructed to facilitate clinical application.

**Results:**

Among 850 patients, 268 (31.5%) were positive for postoperative depression risk. Feature selection identified 13 predictive variables: age, operation time, length of hospital stay, pain score, white blood cell count, albumin, C-reactive protein, CA125, education level, history of depression/anxiety, postoperative insomnia, fatigue, and opioid analgesic use. Among the five models, random forest demonstrated superior performance with an AUC of 0.776 in the validation set, a Brier score of 0.182, sensitivity of 0.771, and an F1 score of 0.792, along with satisfactory calibration and clinical net benefit. SHAP analysis revealed that pain score, postoperative insomnia, albumin level, and opioid use contributed substantially to model predictions. A nomogram based on logistic regression model was constructed for intuitive individual risk assessment.

**Conclusion:**

The machine learning-based prediction models for postoperative depression risk in patients with ovarian cancer demonstrated satisfactory discriminative ability and clinical utility, with random forest model showing optimal performance. A clinical nomogram was additionally constructed to enable individualised and visual risk quantification suitable for bedside application. Together, these tools facilitate early identification of high-risk patients and provide evidence for clinical intervention.

## Introduction

Ovarian cancer represents one of the most lethal malignancies among gynaecologic cancers. Due to insidious early symptoms and the lack of effective screening methods, approximately 80% of patients are diagnosed at advanced stages (International Federation of Gynecology and Obstetrics [FIGO] stage III–IV), requiring cytoreductive surgery combined with platinum-based chemotherapy [1]. Despite the gradual clinical implementation of novel therapeutic strategies including poly (ADP-ribose) polymerase (PARP) inhibitors and immune checkpoint inhibitors, the overall five-year survival rate for patients with ovarian cancer remains limited [2, 3]. Meanwhile, the prolonged, multi-stage treatment process and prognostic uncertainty may exert sustained impacts on patients’ psychological health.

Postoperative depression not only impairs patients’ subjective well-being but also correlates with decreased treatment compliance and adverse clinical outcomes. Previous studies have demonstrated a negative correlation between depressive symptoms and chemotherapy adherence, with patients experiencing more severe depressive symptoms being more likely to discontinue treatment and exhibit poor compliance [4]. Depressive states are frequently accompanied by physiological alterations including hypothalamic–pituitary–adrenal (HPA) axis dysregulation and chronic low-grade inflammation. These changes may influence immune function and tumour microenvironment, thereby associated with poorer survival outcomes [5]. However, in routine clinical practice, identification of postoperative depression often relies predominantly on subjective judgment by healthcare providers or patient self-report, with systematic risk assessment tools not yet widely implemented. This may lead to underestimation or delayed diagnosis of depression [6].

Traditional risk assessment methods, which are primarily based on univariate analysis or simple regression, often struggle to comprehensively capture complex interactive factors. In recent years, machine learning algorithms have demonstrated potential in handling high-dimensional nonlinear relationships and enabling individualised prediction in tumour prognosis and mental health risk prediction, showing superior performance compared to traditional methods in depression and other psychological outcome risk prediction studies [7, 8]. However, machine learning model research specifically targeting postoperative depression risk in ovarian cancer remains relatively scarce. Existing research is often restricted to small sample sizes or single-centre studies, with insufficient exploration of model interpretability.

This study aimed to integrate multidimensional data including demographic characteristics, tumour features, treatment-related factors, perioperative symptoms, and laboratory indicators, employing least absolute shrinkage and selection operator (LASSO) regression and Boruta algorithm for feature selection. Based on the selected features, five machine learning models were developed and compared: logistic regression, random forest, support vector machine, extreme gradient boosting (XGBoost), and neural network. The performance of these models in predicting postoperative depression risk in patients with ovarian cancer was systematically evaluated. Additionally, model decision-making mechanisms were analysed through SHapley Additive exPlanations (SHAP), and a nomogram was constructed to facilitate clinical application, providing scientific evidence for postoperative mental health management in patients with ovarian cancer.

## Methods

### Study Population and Data Source

This study retrospectively enrolled 850 patients with ovarian cancer who underwent surgical treatment at the Department of Gynaecology, West China Second University Hospital, Sichuan University from December 2019 to February 2023. Patient information was obtained from the hospital electronic medical record system, including demographic data, medical history, perioperative clinical parameters, laboratory test results, and follow-up information.

**Fig. 1.**
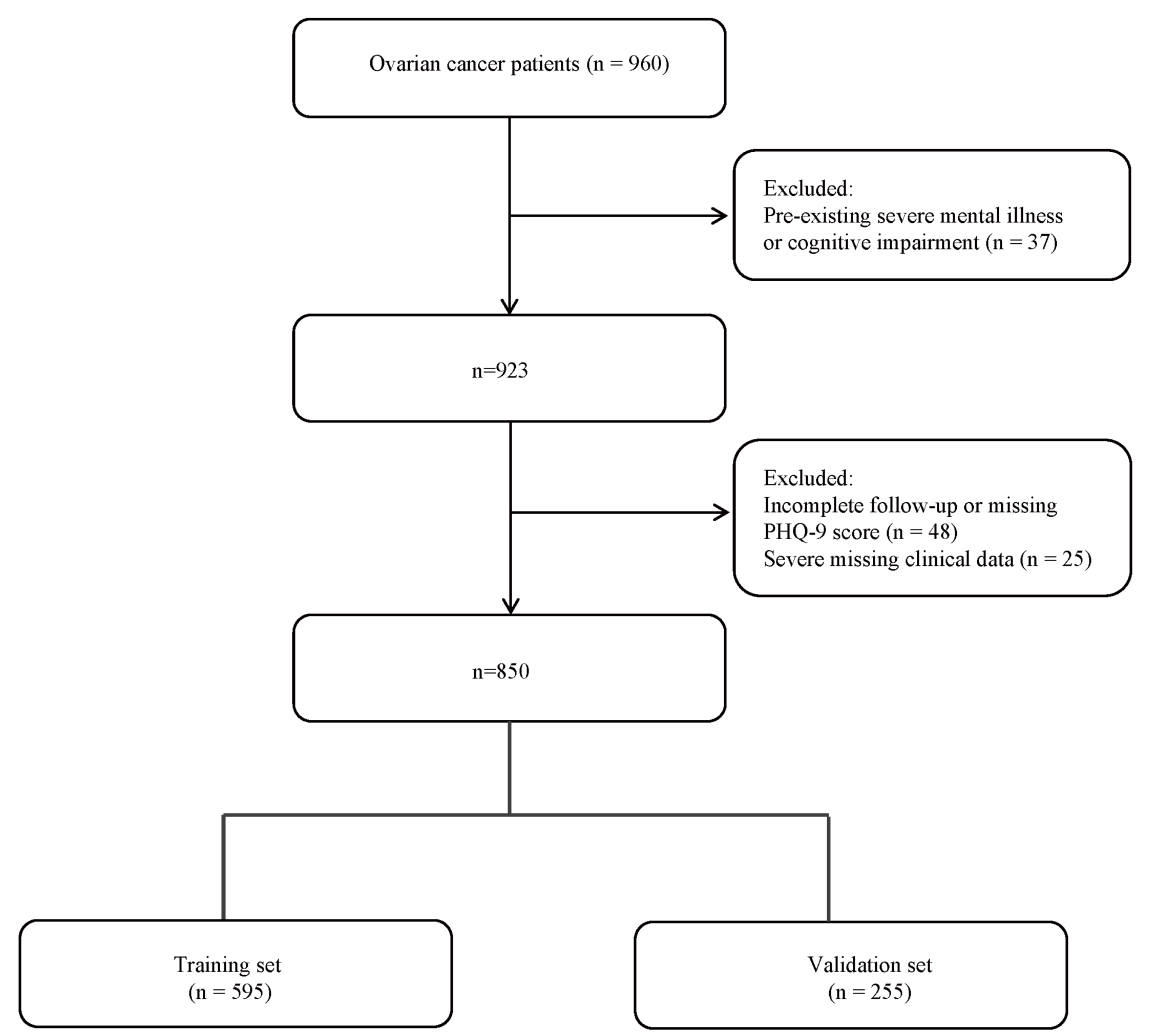
**Flowchart of patient selection and study cohort construction**.

Inclusion criteria were: (1) confirmed diagnosis of ovarian malignancy with surgical treatment, and (2) complete postoperative follow-up information including the Patient Health Questionnaire-9 (PHQ-9) score. Exclusion criteria were: (1) pre-existing severe mental illness or cognitive impairment before surgery, specifically defined as psychotic disorders (e.g., schizophrenia, bipolar disorder with psychotic features) or moderate-to-severe cognitive impairment (e.g., dementia), which could preclude valid self-report completion; patients with a prior history of mild-to-moderate depression or anxiety who were cognitively intact and able to complete assessments were not excluded and were retained in the study cohort, with their psychiatric history recorded as a candidate predictor variable (PsychHistory). (2) Severe missing clinical data that precluded statistical analysis. The detailed patient selection process and reasons for exclusion are presented in Fig. 1.

This study was approved by the Medical Ethics Committee of West China Second University Hospital, Sichuan University (Approval No.: Medical Research 2023 Ethics Approval No. (140), Research No.: K219). All participants provided written informed consent, and the study was conducted in accordance with the Declaration of Helsinki and relevant ethical guidelines.

### Data Collection and Variable Definition

Primary variables included the following categories:

(1) Demographic and sociological characteristics included age (Age, years), body mass index (BMI, kg/m^2^), marital status (MaritalStatus: Married/Divorced/Widowed/Single), education level (Education Level: ≤Junior high school/High school/College or above), residence (Residence: Urban/Rural), and caregiver support (CaregiverSupport: Yes/No).

(2) Lifestyle variables included smoking history (Smoking: Yes/No) and alcohol consumption history (Alcohol: Yes/No). Medical history included history of depression/anxiety (PsychHistory: Yes/No), and history of sleep disorders (SleepDisorderHistory: Yes/No).

(3) Tumour characteristics included FIGO stage (FIGOStage: I/II/III/IV), histological type (Histology: ClearCell/Endometrioid/Mucinous/Serous/Other), and tumour grade (TumorGrade: G1/G2/G3).

(4) Surgical information included surgical approach (SurgicalApproach: Laparoscopy/Open/Robot), surgical intent (SurgicalIntent: interval debulking surgery [IDS]/primary debulking surgery [PDS]/Staging), residual disease (ResidualDisease: Yes/No), bowel resection (BowelResection: Yes/No), operation time (OperationTime, minutes), and intraoperative blood loss (BloodLoss, mL). Postoperative recovery indicators included postoperative complications (PostopComplication: Yes/No), intensive care unit (ICU) admission (ICUAdmission: Yes/No), and length of hospital stay (LengthOfStay, days).

(5) Perioperative status and symptom assessment the following variables. Perioperative performance status was assessed using the Eastern Cooperative Oncology Group (ECOG) Performance Status scale, which ranges from 0 to 5 (0 = fully active, able to carry on all pre-disease performance without restriction; 5 = dead) [9]. In this surgical cohort of patients with ovarian cancer, ECOG scores were limited to 0–3 because patients with ECOG ≥4 (completely disabled or bedridden) were ineligible for cytoreductive surgery according to institutional practice and inclusion criteria, and no ECOG 4 or 5 cases were observed in the retrospective dataset. Pain intensity measured by Numeric Rating Scale (NRS), a 0–10 point self-report scale where 0 indicates no pain and 10 indicates the worst possible pain [10]. Comorbidity burden quantified by Charlson comorbidity index (CharlsonIndex), which includes 19 conditions with weighted scoring based on disease severity, with higher scores indicating greater comorbidity burden [11]. Postoperative symptoms, including insomnia (PostopInsomnia: Yes/No), fatigue (Fatigue: Yes/No), and opioid use (OpioidUse: Yes/No), were assessed daily based on nursing records and patient self-reports during the initial postoperative hospital stay (postoperative days 1–3).

(6) Laboratory indicators included haemoglobin (Hb, g/L), white blood cell count (WBC, ×10^9^/L), neutrophil count (Neutrophils, ×10^9^/L), lymphocyte count (Lymphocytes, ×10^9^/L), platelet count (Platelets, ×10^9^/L), albumin (Albumin, g/L), C-reactive protein (CRP, mg/L), and cancer antigen 125 (CA125, U/mL). Inflammation-related derived indicators included neutrophil-to-lymphocyte ratio (NLR = Neutrophils/Lymphocytes) and platelet-to-lymphocyte ratio (PLR = Platelets/Lymphocytes). Both indices are widely used markers reflecting systemic inflammatory status and have demonstrated prognostic value in various malignancies [12, 13, 14].

(7) Adjuvant treatment information included receipt of adjuvant chemotherapy (AdjuvantChemo: Yes/No), chemotherapy regimen (ChemoRegimen: TC/DC/Other/NoChemo), number of chemotherapy cycles (ChemoCycles: 0–6), bevacizumab use (Bevacizumab: Yes/No), and maintenance therapy type (MaintenanceType: Bev/PARPi/None).

(8) The primary study outcome was postoperative depression risk (Depression: Yes/No). This was determined using the PHQ-9, a validated screening tool for depressive symptoms. To establish a clear temporal sequence and mitigate potential circularity bias, the PHQ-9 was administered after all predictor variables had been collected. Specifically, the PHQ-9 was completed either on the day before hospital discharge (typically postoperative days 5–7) or during the first scheduled postoperative outpatient follow-up visit (within 2–4 weeks after surgery). The PHQ-9 is a 9-item self-report scale with each item scored from 0 to 3 points, yielding a total score ranging from 0 to 27, with higher scores indicating more severe depressive symptoms. In line with established clinical screening thresholds, a PHQ-9 score ≥10 was defined as a positive (Yes) indicator of clinically significant depression risk [15]. It is important to note that a PHQ-9 score ≥10 represents a screening threshold for identifying potential cases rather than a clinical diagnosis of major depressive disorder, which would require comprehensive psychiatric assessment. This threshold has demonstrated high sensitivity and specificity for detecting depression risk in clinical populations.

### Statistical Analysis and Machine Learning Modelling

#### Baseline Characteristics

The baseline characteristics table (Table 1) was generated after handling missing data. The overall proportion of missing data was low (2.44%). Assuming a missing-at-random mechanism, continuous and categorical variables were imputed using the median and mode of the entire sample, respectively. Normality of continuous variables was assessed using the Shapiro-Wilk test. Variables following a normal distribution (*p* ≥ 0.05) are presented as mean ± standard deviation (SD) and compared between groups using the independent samples *t*-test. Variables not following a normal distribution (*p* < 0.05) are presented as median (interquartile range, IQR) and compared using the Mann–Whitney U test. Categorical variables are presented as frequencies and percentages and were compared using Pearson’s Chi-square test or Fisher’s exact test where appropriate. All tests were two-sided, with *p* < 0.05 considered statistically significant.

**Table 1.** **Baseline characteristics of postoperative patients with ovarian cancer**.

Characteristic		Overall (N = 850^1^)	No (N = 582^1^)	Yes (N = 268^1^)	*p*-value^2^
Age		55.21 ± 10.37	55.34 ± 10.58	54.93 ± 9.91	0.6
BMI		23.45 (21.20, 25.50)	23.45 (21.23, 25.90)	23.42 (21.02, 25.00)	0.2
NRS		3.00 (2.00, 4.00)	3.00 (2.00, 4.00)	3.28 (2.43, 4.92)	<0.001
PHQ-9		4.00 (4.00, 4.00)	4.00 (4.00, 4.00)	4.00 (4.00, 4.00)	0.048
Charlson Index		1.00 (0.00, 1.00)	1.00 (0.00, 1.00)	0.00 (0.00, 1.00)	0.2
Operation Time		191.98 (159.00, 224.00)	193.00 (165.00, 224.00)	187.30 (149.00, 223.54)	0.079
Blood Loss		340.00 (224.00, 488.47)	334.91 (234.00, 495.00)	360.00 (204.89, 478.62)	0.6
Length Of Stay		11.87 (9.00, 14.83)	11.30 (8.35, 14.12)	12.64 (9.80, 15.50)	<0.001
Hb		112.64 (104.00, 122.00)	113.00 (104.00, 122.93)	111.33 (103.00, 120.85)	0.2
WBC		6.85 (5.60, 8.01)	6.68 (5.60, 7.70)	7.29 (5.71, 8.50)	<0.001
Neutrophils		4.20 (3.24, 5.00)	4.27 (3.12, 4.96)	4.15 (3.32, 5.13)	0.4
Lymphocytes		1.60 ± 0.39	1.58 ± 0.39	1.64 ± 0.38	0.045
Platelets		261.25 ± 56.86	261.33 ± 56.28	261.08 ± 58.21	>0.9
Albumin		38.20 (36.08, 40.14)	38.20 (36.25, 40.27)	38.10 (35.44, 39.85)	0.022
CRP		4.12 (2.90, 6.53)	4.12 (2.70, 5.90)	4.12 (3.45, 7.61)	0.002
NLR		2.59 (1.97, 3.41)	2.56 (1.91, 3.43)	2.68 (2.09, 3.36)	0.14
PLR		165.60 (136.43, 206.59)	165.71 (137.47, 209.56)	164.11 (133.96, 200.39)	0.6
CA125		319.98 (147.58, 542.60)	319.98 (150.30, 532.80)	319.98 (147.09, 556.77)	0.8
Marital Status					0.9
	Divorced/Widowed	131 (15.41%)	88 (15.12%)	43 (16.04%)	
	Married	601 (70.71%)	411 (70.62%)	190 (70.90%)	
	Single	118 (13.88%)	83 (14.26%)	35 (13.06%)	
Education Level					0.025
	≤JuniorHigh	249 (29.29%)	184 (31.62%)	65 (24.25%)	
	HighSchool	407 (47.88%)	261 (44.85%)	146 (54.48%)	
	College or above	194 (22.82%)	137 (23.54%)	57 (21.27%)	
Residence					0.3
	Rural	321 (37.76%)	212 (36.43%)	109 (40.67%)	
	Urban	529 (62.24%)	370 (63.57%)	159 (59.33%)	
Caregiver Support					0.7
	No	163 (19.18%)	114 (19.59%)	49 (18.28%)	
	Yes	687 (80.82%)	468 (80.41%)	219 (81.72%)	
Smoking					0.3
	No	751 (88.35%)	519 (89.18%)	232 (86.57%)	
	Yes	99 (11.65%)	63 (10.82%)	36 (13.43%)	
Alcohol					>0.9
	No	795 (93.53%)	544 (93.47%)	251 (93.66%)	
	Yes	55 (6.47%)	38 (6.53%)	17 (6.34%)	
Psych History					<0.001
	No	774 (91.06%)	553 (95.02%)	221 (82.46%)	
	Yes	76 (8.94%)	29 (4.98%)	47 (17.54%)	
Sleep Disorder History					0.030
	No	726 (85.41%)	508 (87.29%)	218 (81.34%)	
	Yes	124 (14.59%)	74 (12.71%)	50 (18.66%)	
ECOG					0.5
	0	159 (18.71%)	113 (19.42%)	46 (17.16%)	
	1	391 (46.00%)	257 (44.16%)	134 (50.00%)	
	2	263 (30.94%)	186 (31.96%)	77 (28.73%)	
	3	37 (4.35%)	26 (4.47%)	11 (4.10%)	
Postop Insomnia					<0.001
	No	650 (76.47%)	477 (81.96%)	173 (64.55%)	
	Yes	200 (23.53%)	105 (18.04%)	95 (35.45%)	
Fatigue					<0.001
	No	463 (54.47%)	341 (58.59%)	122 (45.52%)	
	Yes	387 (45.53%)	241 (41.41%)	146 (54.48%)	
Opioid Use					<0.001
	No	734 (86.35%)	529 (90.89%)	205 (76.49%)	
	Yes	116 (13.65%)	53 (9.11%)	63 (23.51%)	
FIGO Stage					0.3
	I	178 (20.94%)	118 (20.27%)	60 (22.39%)	
	II	103 (12.12%)	71 (12.20%)	32 (11.94%)	
	III	416 (48.94%)	296 (50.86%)	120 (44.78%)	
	IV	153 (18.00%)	97 (16.67%)	56 (20.90%)	
Histology					>0.9
	Clear Cell	90 (10.59%)	63 (10.82%)	27 (10.07%)	
	Endometrioid	107 (12.59%)	71 (12.20%)	36 (13.43%)	
	Mucinous	68 (8.00%)	47 (8.08%)	21 (7.84%)	
	Other	50 (5.88%)	34 (5.84%)	16 (5.97%)	
	Serous	535 (62.94%)	367 (63.06%)	168 (62.69%)	
Tumor Grade					0.8
	G1	135 (15.88%)	95 (16.32%)	40 (14.93%)	
	G2	372 (43.76%)	251 (43.13%)	121 (45.15%)	
	G3	343 (40.35%)	236 (40.55%)	107 (39.93%)	
Surgical Approach					0.5
	Laparoscopy	148 (17.41%)	104 (17.87%)	44 (16.42%)	
	Open	651 (76.59%)	440 (75.60%)	211 (78.73%)	
	Robot	51 (6.00%)	38 (6.53%)	13 (4.85%)	
Surgical Intent					0.3
	IDS	285 (33.53%)	187 (32.13%)	98 (36.57%)	
	PDS	491 (57.76%)	346 (59.45%)	145 (54.10%)	
	Staging	74 (8.71%)	49 (8.42%)	25 (9.33%)	
Residual Disease					0.6
	No	567 (66.71%)	392 (67.35%)	175 (65.30%)	
	Yes	283 (33.29%)	190 (32.65%)	93 (34.70%)	
Bowel Resection					0.056
	No	699 (82.24%)	489 (84.02%)	210 (78.36%)	
	Yes	151 (17.76%)	93 (15.98%)	58 (21.64%)	
Postop Complication					0.041
	No	701 (82.47%)	491 (84.36%)	210 (78.36%)	
	Yes	149 (17.53%)	91 (15.64%)	58 (21.64%)	
ICU Admission					0.8
	No	788 (92.71%)	541 (92.96%)	247 (92.16%)	
	Yes	62 (7.29%)	41 (7.04%)	21 (7.84%)	
Adjuvant Chemo					>0.9
	No	135 (15.88%)	93 (15.98%)	42 (15.67%)	
	Yes	715 (84.12%)	489 (84.02%)	226 (84.33%)	
Chemo Regimen					0.4
	DC	118 (13.88%)	88 (15.12%)	30 (11.19%)	
	No Chemo	135 (15.88%)	93 (15.98%)	42 (15.67%)	
	Other	88 (10.35%)	57 (9.79%)	31 (11.57%)	
	TC	509 (59.88%)	344 (59.11%)	165 (61.57%)	
Chemo Cycles					0.6
	0	135 (15.88%)	93 (15.98%)	42 (15.67%)	
	3	83 (9.76%)	57 (9.79%)	26 (9.70%)	
	4	148 (17.41%)	109 (18.73%)	39 (14.55%)	
	5	151 (17.76%)	100 (17.18%)	51 (19.03%)	
	6	333 (39.18%)	223 (38.32%)	110 (41.04%)	
Bevacizumab					0.3
	No	673 (79.18%)	455 (78.18%)	218 (81.34%)	
	Yes	177 (20.82%)	127 (21.82%)	50 (18.66%)	
Maintenance Type					0.2
	Bev	124 (14.59%)	79 (13.57%)	45 (16.79%)	
	None	529 (62.24%)	359 (61.68%)	170 (63.43%)	
	PARPi	197 (23.18%)	144 (24.74%)	53 (19.78%)	

^1^Mean ± SD; median (Q1, Q3); n (%). ^2^Independent samples *t*-test, Wilcoxon rank-sum test, Pearson’s Chi-squared test, or Fisher’s exact test. BMI, body mass index; NRS, Numeric Rating Scale; Hb, haemoglobin; WBC, white blood cell count; CRP, C-reactive protein; NLR, neutrophil-to-lymphocyte ratio; PLR, platelet-to-lymphocyte ratio; ECOG, Eastern Cooperative Oncology Group; IDS, interval debulking surgery; PDS, primary debulking surgery; DC, docetaxel plus carboplatin; TC, paclitaxel plus carboplatin; PARPi, poly(ADP-ribose) polymerase inhibitor.

#### Dataset Partitioning and Variable Selection 

Using Depression as the stratification variable, the data were randomly split into training and validation sets at a ratio of 7:3. Feature selection in the training set was performed using two complementary methods: (1) LASSO logistic regression with 10-fold cross-validation was applied, and variables corresponding to both the λ_min and λ_1se were recorded; and (2) the Boruta algorithm. The intersection of features identified by both methods was defined as the final feature set.

#### Model Construction and Evaluation

Based on the selected feature set, five prediction models were constructed: regularised logistic regression, random forest, support vector machine (radial basis function kernel), XGBoost, and neural network. After model fitting on the training set, predicted probabilities were generated for both training and validation sets. Model performance evaluation included the following aspects: (1) Discriminative ability was assessed using area under the receiver operating characteristic curve (AUC) and Brier score for prediction error; (2) Threshold-related metrics were calculated using a “training set threshold determination and validation set locked evaluation” strategy. Optimal thresholds were determined based on Youden index and fixed specificity targets, calculating accuracy, sensitivity, and specificity; (3) Calibration performance: Platt scaling was applied in the training set to calibrate predicted probabilities, and the calibrated model was then applied to the validation set; (4) Clinical utility: decision curve analysis was performed to evaluate net benefit at different threshold probabilities.

#### Model Interpretation

To enhance clinical interpretability, a multivariable logistic regression model was constructed using the final 13 stable predictive variables selected by LASSO regression and Boruta algorithm for nomogram generation. Continuous variables (Age, LengthOfStay, NRS, WBC, CRP, and CA125) were processed using restricted cubic splines (RCS) with three knots placed at the 10th, 50th, and 90th percentiles of their distributions to capture potential nonlinear relationships with postoperative depression risk. Categorical variables remained as factors. Model fitting employed the lrm() function in the rms package in R (version 4.4.2; R Foundation for Statistical Computing, Vienna, Austria), with internal validation performed using 1000 bootstrap resamples. Regression results including regression coefficients, odds ratios (ORs), and 95% confidence intervals (CI) were used to generate the nomogram. Each variable corresponding to a point score, yielding total score that mapped to individual postoperative depression risk probability. Additionally, SHAP method was employed to interpret feature contributions in the random forest model. Feature importance plots and beeswarm plots were generated to visualise the influence of variables on prediction results. All statistical analyses were conducted in R software (version 4.4.2; R Foundation for Statistical Computing, Vienna, Austria) environment, with *p* < 0.05 considered statistically significant.

## Results

### Baseline Characteristics

This study enrolled 850 postoperative patients with ovarian cancer, including 268 (31.5%) with positive depression risk and 582 (68.5%) with negative depression risk. The mean age of the study population was 55.21 ± 10.37 years, with no statistically significant differences in age or BMI between groups (Table 1). Regarding perioperative clinical characteristics, the depression risk-positive group demonstrated significantly higher pain scores compared with the negative group (median [IQR]: 3.28 [2.43–4.92] vs. 3.00 [2.00–4.00], *p* < 0.001). Preoperative PHQ-9 scores were slightly higher in the positive group (median [IQR]: 4.00 [4.00–4.00] vs. 4.00 [4.00–4.00], *p* = 0.048). The positive group exhibited significantly prolonged length of hospital stay (median [IQR]: 12.64 [9.80–15.50] days vs. 11.30 [8.35–14.12] days, *p* < 0.001). No significant difference was observed in Charlson comorbidity index between groups (median [IQR]: 0.00 [0.00–1.00] vs. 1.00 [0.00–1.00], *p* = 0.2). With respect to laboratory indicators, the positive group showed elevated WBC count (median [IQR]: 7.29 [5.71–8.50] vs. 6.68 [5.60–7.70], *p* < 0.001) and CRP levels (median [IQR]: 4.12 [3.45–7.61] vs. 4.12 [2.70–5.90], *p* = 0.002), with decreased albumin levels (median [IQR]: 38.10 [35.44–39.85] vs. 38.20 [36.25–40.27], *p* = 0.022). The positive group also exhibited higher lymphocyte counts compared with the negative group (mean ± SD: 1.64 ± 0.38 vs. 1.58 ± 0.39, *p* = 0.045). No significant differences were observed in haemoglobin, platelets, NLR, PLR, or CA125 between groups (all *p* > 0.05, Table 1). Among sociodemographic characteristics, education level distribution differed between groups (*p* = 0.025), with a higher proportion of high school education in the positive group (54.48% vs. 44.85%). Marital status, residence, caregiver support, smoking history, and alcohol consumption showed similar distributions (all *p* > 0.05). Regarding medical history, the positive group demonstrated significantly higher proportions of depression/anxiety history (17.54% vs. 4.98%, *p* < 0.001) and sleep disorder history (18.66% vs. 12.71%, *p* = 0.030) compared with the negative group.

For perioperative complications and symptoms, the positive group exhibited significantly higher incidences of postoperative insomnia (35.45% vs. 18.04%), fatigue (54.48% vs. 41.41%), and opioid use (23.51% vs. 9.11%) (all *p* < 0.001). The rate of postoperative complication was also slightly elevated (21.64% vs. 15.64%, *p* = 0.041). No significant differences were observed in ECOG performance status, bowel resection, or ICU admission rate between groups (all *p* > 0.05).

Regarding oncologic characteristics, both groups showed similar distributions in FIGO stage, histological type, tumour grade, surgical approach, and surgical intent (all *p* > 0.05). For treatment-related factors, no significant differences were found in adjuvant chemotherapy proportion, chemotherapy regimen, number of chemotherapy cycles, bevacizumab use, or maintenance therapy type between groups (all *p* > 0.05).

### Feature Selection Process

**Fig. 2.**
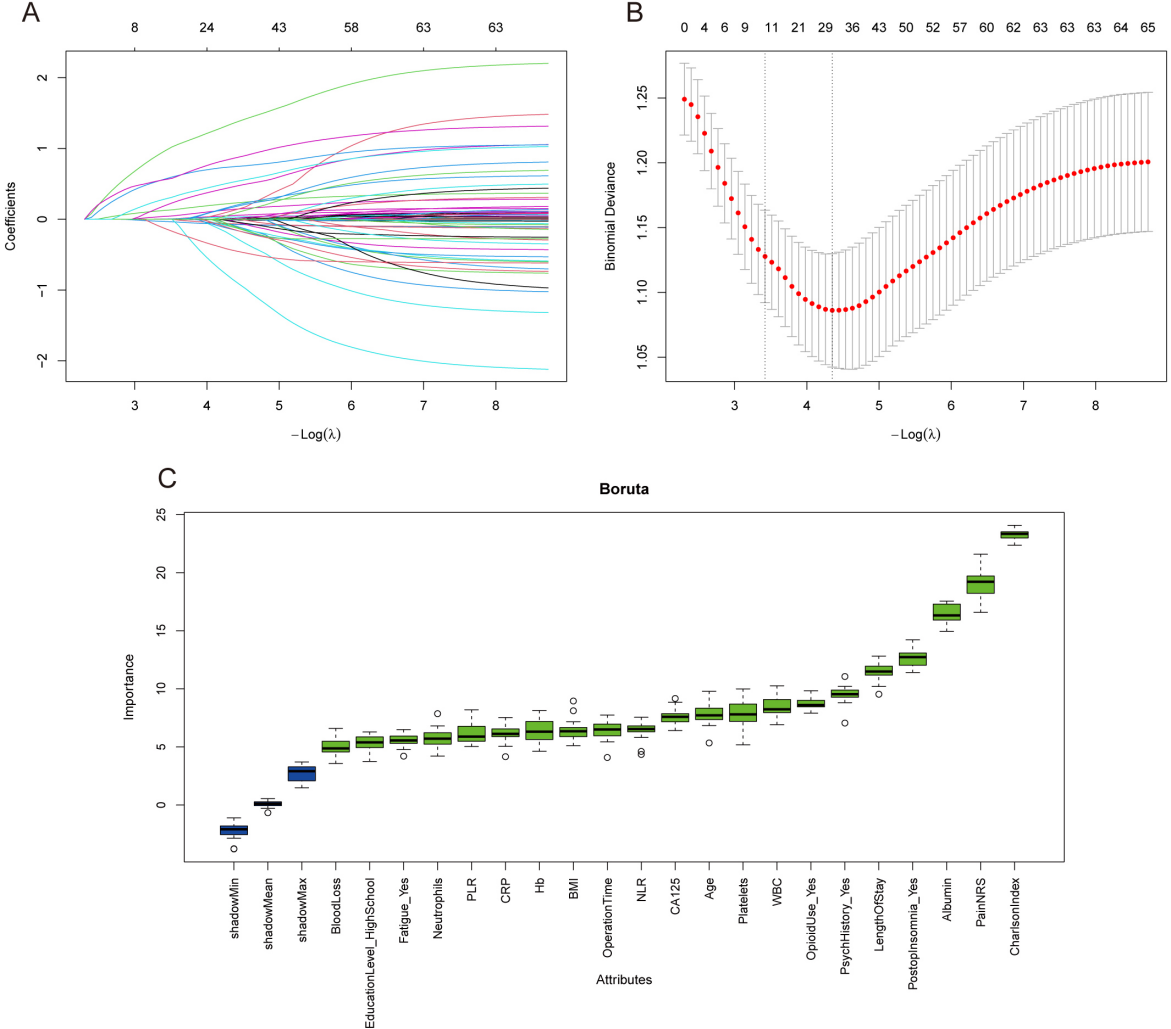
**Feature selection results based on LASSO regression and boruta algorithm**. (A) LASSO regression coefficient path plot, with horizontal axis representing –log(λ) and vertical axis representing regression coefficients of each variable. As the regularisation parameter λ increases, most variable coefficients gradually shrink towards zero. (B) Cross-validation curve of LASSO regression for selecting optimal regularisation parameter λ. Red dots represent cross-validation errors at different λ values, with grey error bars indicating ±1 standard error. Candidate features entering the model were determined based on optimal λ (λ_min). (C) Boruta feature importance analysis results, showing the importance distribution of each variable in the random forest model. Green boxes indicate confirmed important features, yellow boxes indicate tentative features, and blue boxes indicate unimportant features. LASSO, least absolute shrinkage and selection operator.

Feature selection of candidate variables was performed using LASSO regression and Boruta algorithm. In the LASSO regression analysis, the coefficient path plot demonstrated that as the regularisation parameter λ increased, most variable coefficients gradually shrank toward zero (Fig. 2A); the optimal regularisation parameter (λ_min) was determined through cross-validation, under which 31 candidate features associated with postoperative depression risk in ovarian cancer were selected (Fig. 2B). Subsequently, Boruta algorithm was applied to assess the importance of all variables, identifying 21 important features in the full variable analysis (Fig. 2C). By integrating the screening results from both feature selection methods and taking the intersection of features selected by LASSO and Boruta, 13 stable predictive features were finally determined, including age (Age), operation time (OperationTime), length of hospital stay (LengthOfStay), pain score (NRS), white blood cell count (WBC), albumin (Albumin), C-reactive protein (CRP), CA125, education level (Education Level), history of depression/anxiety (PsychHistory), postoperative insomnia (PostopInsomnia), fatigue (Fatigue), and opioid analgesic use (OpioidUse), which were incorporated into subsequent model construction and validation analyses.

### Model Discriminative Ability, Calibration, and Clinical Net Benefit

**Fig. 3.**
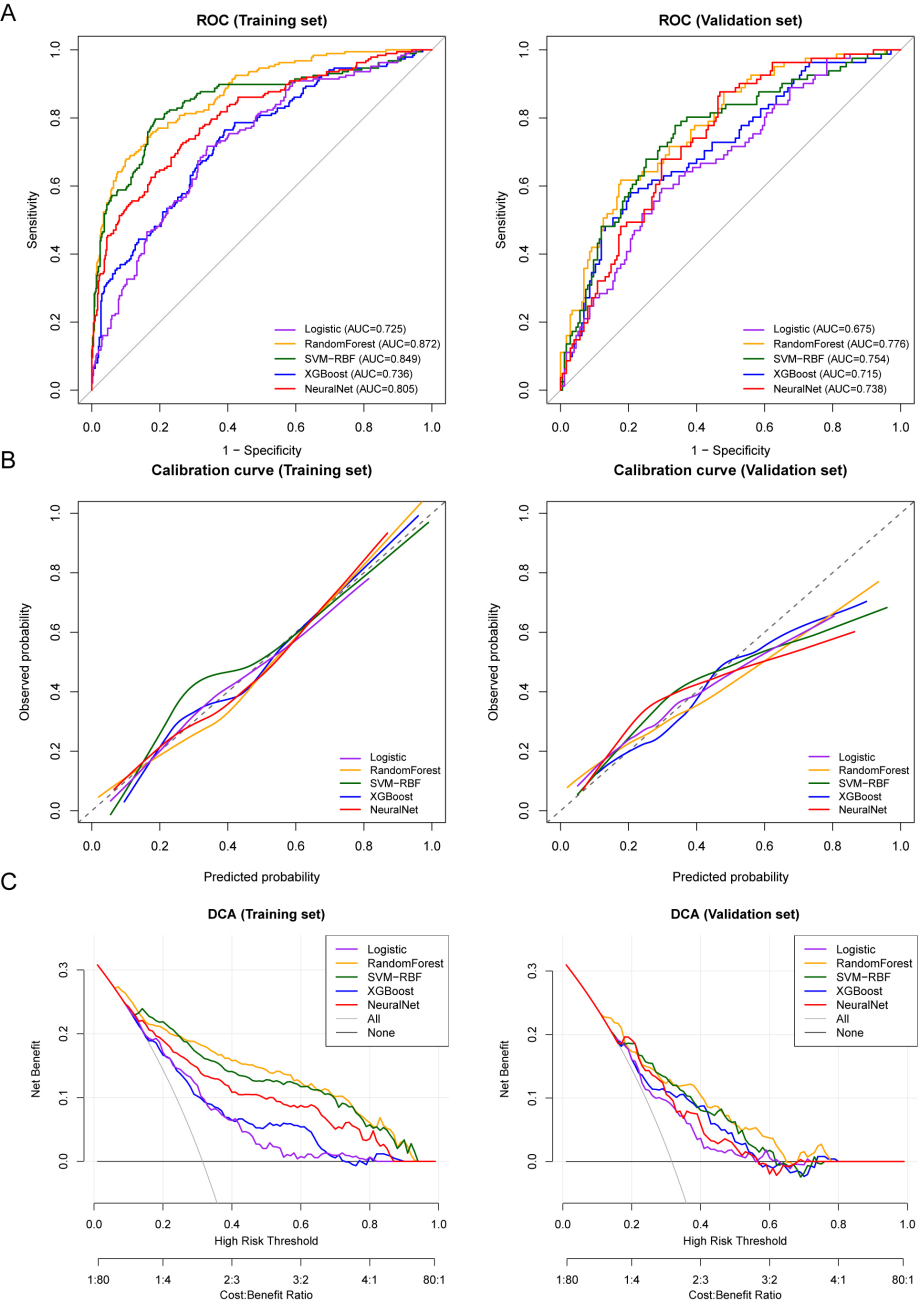
**Discriminative ability, calibration, and clinical net benefit of different machine learning models for predicting postoperative depression risk in patients with ovarian cancer**. (A) Receiver operating characteristic (ROC) curves for evaluating discriminative ability of each model in training and validation sets, with area under the curve (AUC) reflecting the model’s ability to distinguish between depressed and non-depressed patients. (B) Calibration curves comparing consistency between model-predicted probabilities and actual observed probabilities, with dashed line representing ideal perfect calibration. (C) Decision curve analysis (DCA) for evaluating clinical net benefit of different models at various high-risk thresholds, compared with “treat all” and “treat none” strategies.

The comparative predictive performance of different machine learning models in training and validation sets is shown in Tables 2,3, and Fig. 3. In the training set (Table 2), the random forest model demonstrated optimal comprehensive predictive performance among all compared models, with the highest discriminative ability (AUC = 0.872) and the lowest Brier score (0.149), suggesting its advantage in probability prediction accuracy. Support vector machine and neural network models also exhibited satisfactory discriminative ability and classification performance in the training set, whereas XGBoost and logistic regression models showed relatively lower overall predictive performance. Regarding classification metrics, the random forest model achieved high sensitivity (0.838) and F1 score (0.857) in the training set, while maintaining relatively balanced specificity, indicating good capability in identifying high-risk depression patients.

**Table 2.** **Comparison of predictive performance among different models in the training set**.

Model	AUC	Brier	Sensitivity	Specificity	F1
Logistic	0.725	0.201	0.661	0.717	0.738
Random Forest	0.872	0.149	0.838	0.743	0.857
SVM-RBF	0.849	0.156	0.813	0.797	0.853
XGBoost	0.736	0.196	0.609	0.765	0.710
Neural Network	0.805	0.181	0.732	0.727	0.788

AUC, receiver operating characteristic curve.

**Table 3.** **Comparison of predictive performance among different models in the validation set**.

Model	AUC	Brier	Sensitivity	Specificity	F1
Logistic	0.675	0.199	0.634	0.642	0.705
Random Forest	0.776	0.182	0.771	0.617	0.792
SVM-RBF	0.754	0.181	0.691	0.716	0.759
XGBoost	0.715	0.197	0.486	0.728	0.603
Neural Network	0.738	0.202	0.703	0.630	0.750

In the validation set (Table 3), all models showed decreased predictive performance compared with the training set, although the overall trend remained consistent. The random forest model continued to demonstrate highest discriminative ability in the validation set (AUC = 0.776) and maintained a low Brier score (0.182), indicating satisfactory generalisation ability and prediction stability. Support vector machine and neural network models also exhibited good predictive performance in the validation set (AUC of 0.754 and 0.738, respectively), though their comprehensive classification performance was slightly inferior to that of the random forest model. In contrast, the logistic regression and XGBoost models showed relatively lower AUC and F1 scores in the validation set. From a classification performance perspective, the random forest model achieved high sensitivity (0.771) and an F1 score of 0.792 in the validation set, demonstrating certain advantages in identifying high-risk patients. Despite relatively lower specificity, it overall presented relatively balanced classification performance.

ROC curve results further validated the above findings, with the random forest model’s ROC curve demonstrating superior discriminative performance compared with other models in both training and validation sets (Fig. 3A). Model calibration performance is shown in Fig. 3B, with the random forest model demonstrating relatively good calibration consistency in both training and validation sets. Decision curve analysis results showed that across a wide range of threshold probabilities, the random forest model achieved higher clinical net benefit in both training and validation sets (Fig. 3C).

### Feature Importance and Interpretability Analysis of Random Forest Model

**Fig. 4.**
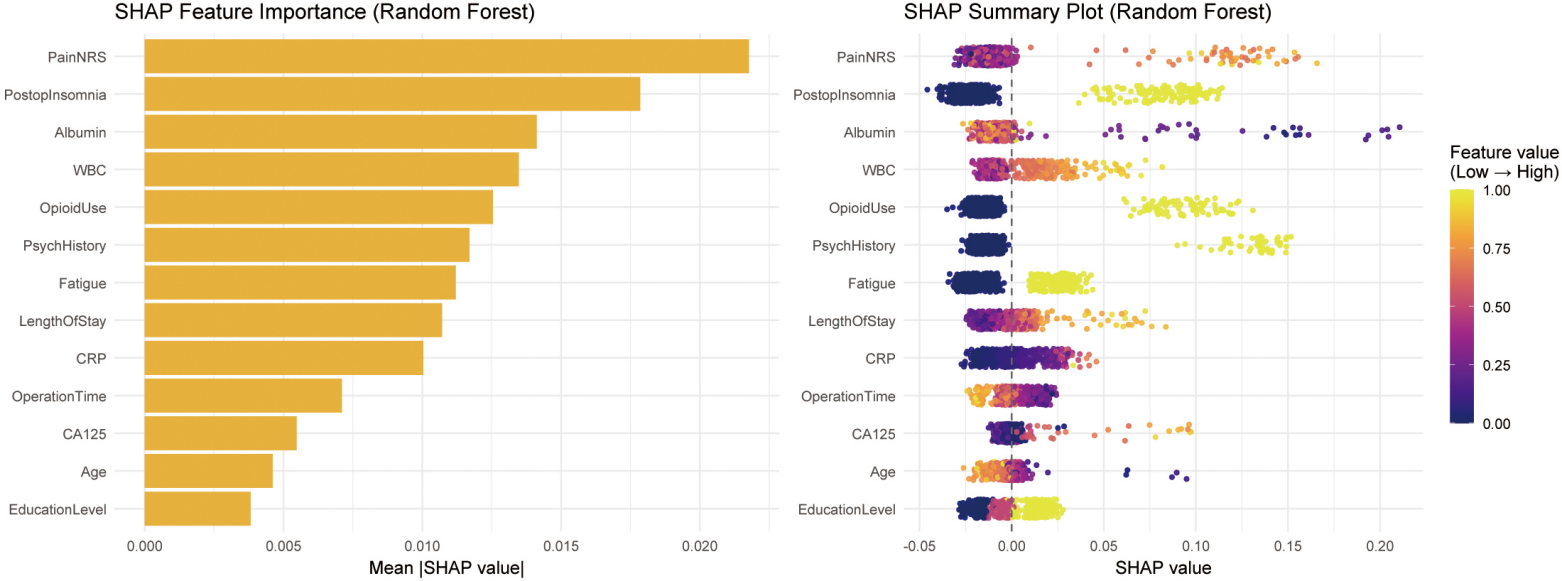
**SHAP feature importance and summary analysis of random forest model**. The left panel displays mean absolute SHAP values of each feature in the random forest model, quantifying the relative importance of different variables for model prediction; the right panel shows the SHAP summary plot, presenting the distribution of SHAP values across all samples and their directional impact on prediction results. Point colour represents feature value magnitude (from low to high), horizontal axis represents SHAP value, with positive values indicating the feature increases postoperative depression risk and negative values indicating decreased risk.

To interpret the prediction mechanism of the random forest model, SHAP method was employed for interpretability analysis (Fig. 4). SHAP-based feature contribution ranking revealed that NRS contributed most substantially to model prediction, followed by PostopInsomnia, Albumin, WBC, and OpioidUse. Additionally, PsychHistory, Fatigue, LengthOfStay, and inflammation-related indicators (such as CRP) also contributed to model prediction. In contrast, age, education level, and CA125 showed relatively smaller contributions.

SHAP summary plot further revealed the directional influence of each variable on prediction results. Higher pain scores, postoperative insomnia, opioid analgesic use, history of psychiatric illness, and fatigue status typically corresponded to positive SHAP values, suggesting their association with model prediction of higher postoperative depression risk; whereas higher albumin levels primarily associated with negative SHAP values, indicating their protective effect against depression risk. Overall, SHAP analysis demonstrated that the random forest model primarily relied on perioperative symptoms and previous mental health status when predicting postoperative depression risk in ovarian cancer, while integrating biological information such as inflammation and nutritional status.

### Construction and Validation of Nomogram for Postoperative Depression Risk Prediction

**Fig. 5.**
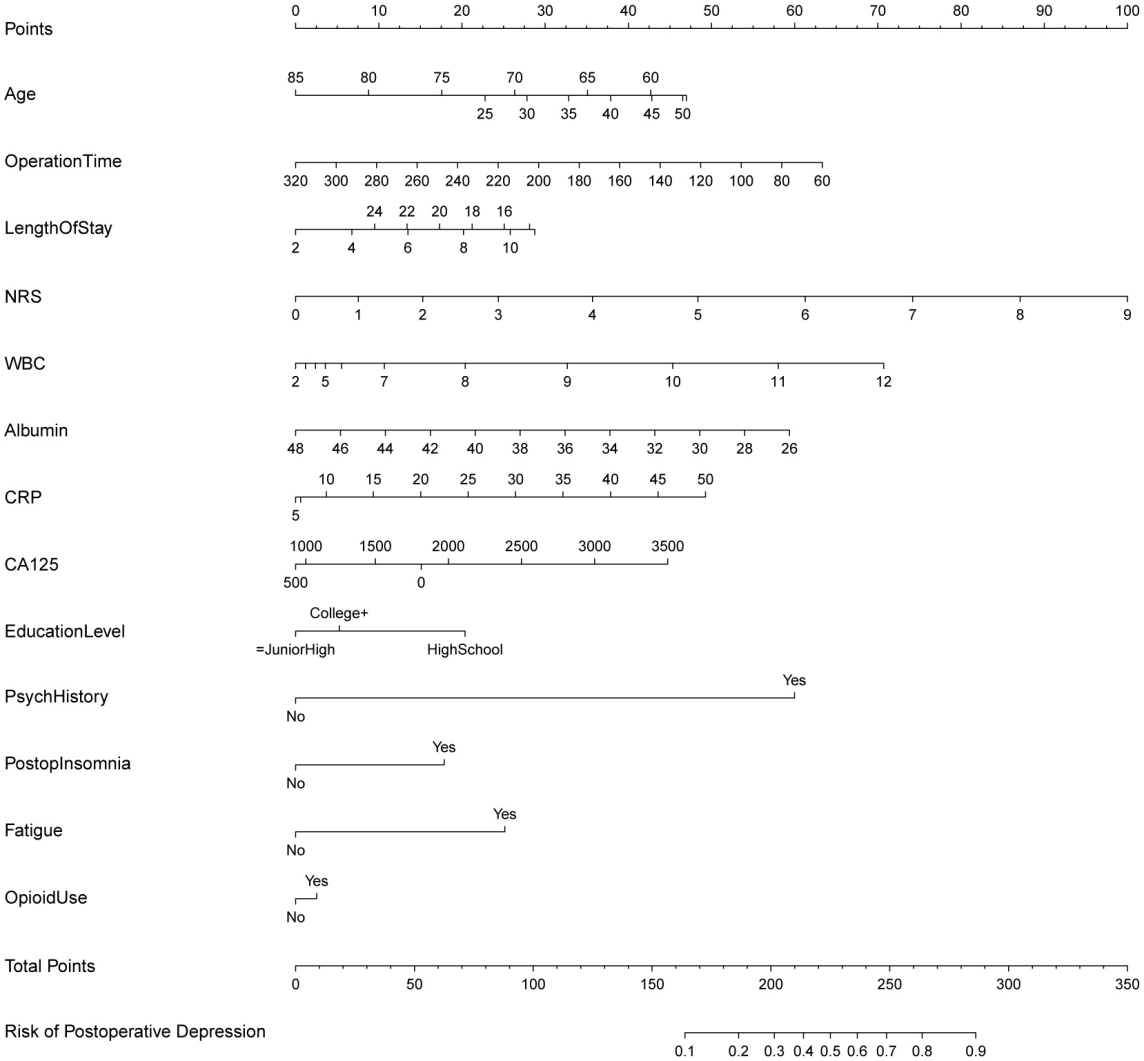
**Nomogram for predicting postoperative depression risk in patients with ovarian cancer**. This nomogram was constructed based on logistic regression model for predicting postoperative depression risk in patients with ovarian cancer. Each predictive variable corresponds to a scale line, with corresponding point values above. Based on patient-specific values, corresponding points are read from each variable scale line and summed to obtain total points, which are then mapped on the bottom scale to predicted probability of postoperative depression (Risk of Postoperative Depression).

A multivariable logistic regression model was constructed using the 13 predictors selected by the LASSO regression and the Boruta algorithm for the development of a clinical nomogram (Fig. 5). To capture potential nonlinear relationships, continuous variables (Age, OperationTime, LengthOfStay, NRS, WBC, Albumin, CRP, CA125) were modelled using RCS with three knots (placed at the 10th, 50th, and 90th percentiles). The odds ratios (ORs) presented in Table 4 represent the overall effect of each variable on postoperative depression risk, derived from the fitted RCS model.

**Table 4.** **Odds ratios (ORs), 95% confidence intervals (CIs), and *p* values of the multivariable logistic regression model used for the nomogram**.

Variable	OR (95% CI)	*p* value
Age	1.22 (0.60–2.39)	0.340
OperationTime	0.57 (0.37–0.90)	0.015
LengthOfStay	1.99 (0.70–5.67)	0.197
NRS	1.72 (0.65–4.52)	0.273
WBC	1.11 (0.34–3.60)	0.864
Albumin	0.64 (0.44–0.93)	0.020
CRP	0.95 (0.32–2.82)	0.931
CA125	0.48 (0.16–1.44)	0.189
Education Level = College+	1.21 (0.53–2.74)	0.652
Education Level = HighSchool	2.08 (1.05–4.13)	0.037
PsychHistory = Yes	8.62 (2.87–25.90)	<0.001
PostopInsomnia = Yes	1.90 (0.97–3.71)	0.061
Fatigue = Yes	2.47 (1.37–4.44)	0.003
OpioidUse = Yes	1.10 (0.47–2.58)	0.835

Note: Continuous variables (Age, OperationTime, LengthOfStay, NRS, WBC, Albumin, CRP, CA125) were modelled using restricted cubic splines with three knots. The ORs presented represent the overall association of each variable with postoperative depression risk, derived from the combined spline terms.

The model identified several independent predictors. A history of depression or anxiety was the strongest predictor of increased risk (OR = 8.62, 95% CI: 2.87–25.90, *p* < 0.001). The presence of postoperative fatigue (OR = 2.47, 95% CI: 1.37–4.44, *p* = 0.003) and a high school education level (vs. ≤junior high; OR = 2.08, 95% CI: 1.05–4.13, *p* = 0.037) were also significantly associated with higher risk. Lower serum albumin levels were associated with significantly increased risk (OR = 0.64, 95% CI: 0.44–0.93, *p* = 0.020). Operation time exhibited a nonlinear association with postoperative depression risk, with longer procedures associated with lower overall odds of depression (OR = 0.57, 95% CI: 0.37–0.90, *p* = 0.015). The effects of other continuous predictors, including Age, NRS, and CRP were not statistically significant in the multivariable model (all *p* > 0.05, Table 4).

The nomogram (Fig. 5) visually translates these predictors, incorporating their potentially nonlinear relationships, into a points-based scoring system. The total points correspond to an individual patient’s predicted probability of postoperative depression.

### Sensitivity Analysis

To address potential concerns regarding the exclusion of tumour-related variables, a sensitivity analysis was conducted by forcibly incorporating five oncologic variables (FIGO stage, histological type, tumour grade, residual disease status, and surgical intent) into the optimal random forest model alongside the original 13 predictors.

As shown in Table 5, the inclusion of these tumour variables resulted in minimal changes in model performance. The validation set AUC showed a negligible increase from 0.776 to 0.778 (Δ = +0.002), while the Brier score—reflecting overall prediction error—increased slightly from 0.182 to 0.189 (Δ = +0.007). Although sensitivity improved from 0.771 to 0.781 (Δ = +0.010), this gain was offset by a reduction in specificity (0.617 to 0.580, Δ = –0.037). The F1 score, balancing sensitivity and precision, showed a marginal improvement from 0.792 to 0.798 (Δ = +0.006).

**Table 5.** **Sensitivity analysis comparing the performance of random forest models before and after inclusion of tumour-related variables**.

Performance metric	Primary model	Extended model
Number of features	13	18 (+5)
Training AUC	0.872	0.880 (+0.008)
Validation AUC	0.776	0.778 (+0.002)
Training Brier score	0.149	0.161 (+0.012)
Validation Brier score	0.182	0.189 (+0.007)
Sensitivity (validation)	0.771	0.781 (+0.010)
Specificity (validation)	0.617	0.580 (–0.037)
F1 score (validation)	0.792	0.798 (+0.006)

Note: Primary model includes 13 variables selected by the intersection of LASSO regression and Boruta algorithm. Extended model includes the same 13 variables plus five tumour-related variables: FIGO stage, histological type, tumour grade, residual disease status, and surgical intent. Values in parentheses represent differences between extended and primary models (extended – primary). All performance metrics were calculated on the validation set using the Youden index-optimised threshold.

These results demonstrated that tumour-related variables did not meaningfully enhance the predictive performance for early postoperative depression risk. The minimal performance changes (all |Δ| <0.04) fall within the range of random variation and do not represent clinically significant improvement, thereby supporting the robustness of our feature selection approach.

## Discussion

This study constructed machine learning-based models to predict postoperative depression risk in patients with ovarian cancer. Among the evaluated models, the random forest model achieved the best performance, with an AUC of 0.776, demonstrating satisfactory discriminative ability. The observed prevalence of postoperative depression risk was 31.5%. SHAP interpretability analysis further indicated that pain intensity, postoperative insomnia, inflammation and nutrition status indicators, and previous mental health history represented key features influencing model prediction. These findings provide a quantitative tool for precise identification of high-risk perioperative patients.

### Methodological Explanation of Postoperative Depression Prevalence

The prevalence of postoperative depression risk observed in this study (31.5%) was significantly higher than the meta-analysis result by Watts* et al*. (12.71%) [16]. This discrepancy may primarily relate to differences in assessment tools, population characteristics, and inclusion criteria. This study employed PHQ-9 ≥10 as the criterion for postoperative depression risk positivity, a threshold with high sensitivity and specificity (both approximately 0.85) [17], though it essentially remains a self-report screening tool. In contrast, assessment methods used in studies included by Watts* et al*. [16] were more diverse, including structured clinical interviews and different types of self-report scales, with notable heterogeneity in diagnostic criteria and cut-off values. Previous studies have demonstrated that self-report scales often produce higher prevalence estimates compared with clinical interviews, which may partially amplify the depression risk-positive proportion in our study. Additionally, differences in assessment timepoints may significantly impact results [18]. Our study focused on depression risk during the perioperative and early postoperative period, whereas “post-treatment” defined in previous studies encompassed a broader time range. Population characteristic differences also warrant attention. The participants in this study originated from a single-centre cohort in southwestern China, with a depression prevalence of 31.5% observed in our cohort, whereas Liu* et al*. [19] reported prevalence rates of depression (47.0%) and anxiety (51.5%) symptoms in Chinese patients with ovarian cancer, notably higher than those reported in many Western studies [16, 19, 20, 21]. This discrepancy may relate to cultural background (such as emotional expression patterns and illness perception), healthcare system characteristics, and social support structures. Recent updated meta-analyses further revealed pooled prevalence rates of depression and anxiety in patients with ovarian cancer reaching 27% and 33%, respectively [21], higher than Watts* et al*.’s [16] 2015 estimates, reflecting evolution in research methods and included population composition over time.

### Model Construction and Performance Evaluation

Through the dual screening strategy combining LASSO regression and Boruta algorithm, this study finally identified 13 stable predictive variables. This strategy, taking the intersection of both methods, effectively reduced risks of overfitting or missing important variables that might arise from a single method, enhancing feature selection robustness [22].

Among the five compared machine learning models, random forest demonstrated the best overall performance, with an AUC of 0.776 and a Brier score of 0.182 in the validation set. This discriminative performance was comparable to the depression risk prediction model for cancer patients recently developed by de Hond* et al*. [23] (LASSO logistic regression model, AUC = 0.74), though the random forest model in this study showed greater advantage in sensitivity. In the depression prediction model for middle-aged and elderly cancer patients constructed by Xiao* et al*. [24] based on the China Health and Retirement Longitudinal Study (CHARLS) cohort, the random forest model similarly achieved optimal predictive efficacy (AUC = 0.774), highly consistent with findings from the current study. Sensitivity analysis forcing inclusion of tumour-related variables demonstrated minimal performance changes, suggesting that acute perioperative stressors, rather than oncologic characteristics, dominate depression risk during the early postoperative assessment window.

The satisfactory predictive performance of random forest algorithm in this study may be closely related to its methodological characteristics. As an ensemble learning method, random forest effectively captures potential nonlinear relationships and high-order interaction effects among variables through constructing and aggregating multiple decision trees [25], thereby enhancing model adaptability to complex data structures. Meanwhile, this algorithm demonstrates strong robustness to outliers and random noise, maintaining stable performance under measurement errors and distributional skewness commonly encountered in clinical data. Additionally, the built-in variable importance assessment mechanism in random forest provides intuitive basis for identifying key predictive factors, contributing to enhanced model interpretability [25, 26, 27]. Previous studies have also demonstrated that in contexts such as tumour prognosis prediction, when data exhibit obvious nonlinear features or violate proportional hazards assumptions, random forest models often outperform traditional Cox regression models in predictive performance [28].

Notably, although the random forest model’s specificity (0.617) in the validation set was relatively modest, its higher sensitivity better aligns with the primary purpose of screening tools. In the clinical context of postoperative depression risk assessment, the screening phase typically emphasises reducing missed identification of high-risk patients to enable timely further evaluation and intervention; therefore, prediction models with high sensitivity may have potential clinical value in the initial screening stage [29].

### Kynurenine Pathway Mechanism of Pain-Inflammation-Depression

Pain score as the most important predictor of postoperative depression risk may reflect the critical role of pain-induced inflammatory and metabolic alterations in the development of depression. Notably, pain in patients with ovarian cancer is not solely attributable to surgical trauma. Platinum-based chemotherapy, the cornerstone of first-line ovarian cancer treatment, can induce chemotherapy-induced peripheral neuropathy in up to approximately 68% of patients shortly after treatment [30]. This neuropathy may persist into the perioperative period, compound postoperative nociceptive pain, and amplify peripheral neuroinflammatory signalling. Surgery-related pain and tissue injury can activate peripheral immune responses, subsequently affecting central nervous function through the tryptophan-kynurenine (KYN) metabolic pathway [31]. Surgery-related pain and tissue injury activate peripheral immune responses, leading to increased release of pro-inflammatory cytokines such as IL-6 and TNF-α. These inflammatory signals can be transmitted to the central nervous system through humoral and neural pathways, inducing microglial activation and amplifying neuroinflammatory responses [32]. Sustained inflammatory stimulation can induce pro-inflammatory cytokines (such as IFN-γ, TNF-α) to significantly enhance indoleamine-2,3-dioxygenase (IDO) activity, shifting tryptophan (TRP) metabolism more toward the KYN pathway rather than 5-hydroxytryptamine (5-HT) synthesis. Inflammation-induced IDO activation not only elevates generation of KYN and its downstream metabolites but also reduces TRP substrate available for 5-HT synthesis, thereby contributing to neurobiological alterations associated with inflammation-related depression [33, 34]. Furthermore, KYN is further metabolised in the central nervous system to quinolinic acid (QUIN), which can enhance glutamatergic excitotoxicity through activating N-methyl-D-aspartate receptors, which is considered to play an important role in inflammation-related depression development [35]. Animal experimental studies have also demonstrated that intervening in KYN metabolism to QUIN can attenuate inflammation-induced depressive-like behaviours, suggesting central KYN metabolites possess critical regulatory significance in this process [36].

In this study, the depression risk-positive group exhibited elevated CRP and WBC levels with decreased albumin levels. This inflammation–nutrition imbalance phenotype shows directional consistency with the aforementioned peripheral-central inflammatory pathways, though specific molecular mechanisms require further research validation. Notably, approximately 24% of depression risk-positive patients used opioid analgesics, which may not only reflect more severe pain but also suggest opioids might potentially influence depression risk through immune modulation or HPA axis interference [37].

### Bidirectional Causality and Neural Circuit Dysfunction Between Insomnia and Depression

Postoperative insomnia incidence in the depression risk-positive group (35.45%) was nearly twice that of the negative group (18.04%), making insomnia the second most important predictive factor in our model. Meta-analyses of multiple prospective cohort studies have shown that insomnia is significantly associated with future depression risk, approximately doubling future depression risk (RR = 2.27, 95% CI: 1.89–2.71) [38]. Neuroimaging studies have revealed the core neural circuit shared between insomnia and depression, characterised by dysfunctional between connectivity between the prefrontal cortex and the amygdala [39]. Sleep deprivation leads to decreased prefrontal metabolic activity and enhanced amygdala activity, with top-down emotional regulation capacity collapsing and pathologically enhanced sensitivity to negative stimuli. In patients with ovarian cancer, bilateral oophorectomy induces abrupt estrogen withdrawal, which likely contributes to the poorer sleep quality and higher insomnia risk observed in this population, consistent with findings in surgical versus natural menopause [40]. Meanwhile, platinum-based and other neurotoxic chemotherapeutic regimens have been shown to impair sleep quality through treatment-related neurotoxicity and gastrointestinal adverse effects in cancer patients [41]. Additionally, sleep disturbances, including insomnia, can activate the sympathetic nervous system and pro-inflammatory cytokine pathways, contributing to depressive symptomatology and forming a vicious cycle of insomnia, inflammation, and depression [42]. Cho* et al*. [43] found that in depressed patients, sleep disturbances correlated with altered KYN pathway metabolic balance, manifesting as sleep problems associated with decreased neuroprotective metabolic ratios (such as KynA/QA), suggesting sleep disturbances may participate in depression pathology through affecting KYN pathway branch balance. In the present study, 18.66% of depression risk-positive patients had history of sleep disorders, suggesting these patients’ sleep regulatory systems were already in a vulnerable state, with surgical stress more easily pushing them toward decompensation. From an intervention perspective, cognitive behavioural therapy for insomnia has been proven to improve sleep quality in cancer patients and alleviate depressive symptoms [44], suggesting early management of postoperative insomnia may be an effective entry point for depression prevention.

### Predictive Value of Albumin as Inflammatory Marker

Albumin was one of the few factors demonstrating protective effects (OR = 0.64), though its role likely reflects inflammatory status rather than simple malnutrition [45]. During acute inflammatory responses, humoral and cellular-level changes associated with pro-inflammatory signals can remarkably increase capillary permeability, promoting albumin extravasation from intravascular to interstitial space. This process expands the distribution volume of albumin and shortens its half-life, ultimately leading to decreased serum albumin levels, reflecting inflammation’s profound impact on albumin metabolic balance [45]. In advanced ovarian cancer, malignant ascites reflects high tumour burden and an inflammatory tumour microenvironment, characterised by protein-rich exudative fluid and elevated pro-inflammatory cytokines such as interleukin-6. Consequently, hypoalbuminemia in advanced disease represents a composite marker integrating tumour burden, exudative protein accumulation, and systemic inflammation [46]. In the present study, the albumin difference between groups was only 0.1 g/L with values remaining within normal range; this mild decrease more likely represents sustained low-grade inflammation rather than severe nutritional deficiency. Albumin, as the most abundant non-enzymatic antioxidant in plasma, directly scavenges free radicals and inhibits oxidation reactions through its free sulfhydryl groups and capacity to bind oxidative molecules. Decreased albumin levels weaken the body’s antioxidant defence system [47, 48]. Meanwhile, depression pathogenesis closely relates to activation of oxidative and nitrosative stress pathways and overall decline in antioxidant capacity, mechanisms collectively suggesting antioxidant defence impairment may play an important role in depression development and progression [49]. Elevated CRP and WBC in the present study further supported the role of inflammation in postoperative depression. Although not statistically significant in multivariable analysis, SHAP analysis demonstrated they still exerted predictive effects through interactions with other features.

### Potential Clinical Interpretation of the Association Between Operative Time and Depression Risk

Notably, longer operation time was associated with lower odds of postoperative depression (OR = 0.57, 95% CI: 0.37–0.90, *p* = 0.015). This finding may appear counterintuitive, but becomes interpretable when operation time is considered as a surrogate for the extent of cytoreduction. In ovarian cancer, the prognostic value of thorough debulking is well established: each 10% increase in maximal cytoreduction corresponds to a 5.5% increase in median survival time among patients receiving platinum-based chemotherapy [50], and each 10% increase in the proportion achieving no gross residual disease independently contributes a 2.3-month gain in median survival of the cohort [51]. Patients who undergo more extensive resection may therefore develop a stronger perception of treatment efficacy, which in turn attenuates psychological distress, consistent with evidence that curability belief inversely correlates with depression in advanced cancer patients [52]. Importantly, the SOCQER-2 multicentre study found no association between high-complexity cytoreductive surgery and greater depression or anxiety at 12 months [53], suggesting that surgical extent does not worsen mood outcomes. It should nonetheless be acknowledged that operation time is an imperfect proxy, potentially reflecting intraoperative complexity rather than resection success, and residual confounding cannot be excluded.

### Strong Predictive Effect of Previous Mental Health History and Its Mechanisms

PsychHistory was the strongest independent predictor of postoperative depression risk (OR = 8.62), a finding highly consistent with numerous previous studies. Bouras* et al*. [54] found in patients after esophagogastric cancer resection that a history of psychiatric illness was an important predictor of postoperative depression/anxiety (OR = 6.73, 95% CI: 4.25–10.64). The European Society for Medical Oncology clinical practice guideline for anxiety and depression in cancer patients [7] explicitly lists history of mood disorders as an individual risk factor for depression. The recurrence rate after an initial depressive episode is approximately 50%, increasing to 90% after three episodes, demonstrating a “recurrence facilitation” phenomenon [55]. According to the kindling (stress sensitisation) theory originally proposed by Post [56], as patients with depressive disorders repeatedly experience episodes over the course of illness, their sensitivity to life stress gradually increases, with new episodes more easily triggered even under lower-intensity stress, reflecting decreased recurrence threshold. This theoretical framework explains why repeated stress (such as major life events, cancer diagnosis, and surgery) more easily triggers subsequent depression recurrence [56]. From a clinical practice perspective, patients with ovarian cancer who have a history of psychiatric illness should receive more intensive psychological assessment and necessary preventive interventions during the perioperative period, including early psychiatric consultation and psychotherapy.

### Clinical Application and Study Limitations

Based on identified risk factors, a pragmatic intervention framework can be proposed. High-risk patients—particularly those with prior psychiatric history (OR = 8.62), postoperative fatigue (OR = 2.47), severe pain, or insomnia—should receive enhanced monitoring and targeted interventions. Evidence-based approaches include cognitive behavioural therapy for insomnia, which has demonstrated efficacy in reducing both sleep disturbance and depressive symptoms in cancer populations [57], and optimised multimodal analgesia addressing pain-inflammation pathways. For patients with multiple risk factors, early psychiatric consultation may be warranted. From a resource-allocation perspective, risk-stratified interventions concentrating on high-risk individuals may improve cost-effectiveness compared with universal approaches. Systematic reviews and meta-analyses indicate that collaborative care interventions significantly improve depressive outcomes in cancer patients, while economic evaluations of specific collaborative care programmes suggest favourable cost-effectiveness compared with usual care [58]. However, formal economic evaluation of model-guided interventions in ovarian cancer populations remains necessary.

Although the random forest model demonstrated optimal predictive performance, its structural complexity and limited interpretability may constrain its direct bedside application. Therefore, this study also constructed a nomogram model based on logistic regression, seeking relative balance between predictive performance and clinical usability. Further introduction of SHAP methodology for interpretability analysis of the random forest model enabled quantification of relative contributions of predictive variables to individual risk estimation, helping reveal model decision-making logic and thereby enhancing clinician’s understanding and trust in model outputs [59]. However, a critical “last mile” exists from prediction model to clinical application: external validation is needed to assess generalisation, and prospective implementation studies are required to evaluate whether these models can change clinical decision-making and improve patient outcomes.

This study has several limitations. First, the retrospective single-centre study design with internal validation only limits causal inference and result generalisability. No external (temporal or geographical) validation was performed; therefore the generalisability of the prediction models requires further confirmation in independent multicentre cohorts. Second, a PHQ-9 score ≥10 identifies moderate to severe depressive symptom risk rather than standardised psychiatric diagnosis, which may introduce potential heterogeneity in symptom phenotype and course among the study population. Third, some important psychosocial factors (such as coping styles and social support quality) were not included in analysis, potentially leading to residual confounding. Although broader proxies (such as caregiver support and residence type) were among initial candidate variables, they were not retained in the final model through feature selection process. Fourth, this study only measured non-specific inflammatory markers such as CRP and WBC, without directly detecting pro-inflammatory cytokines or KYN pathway-related metabolites, thereby limiting in-depth exploration of underlying biological mechanisms. Fifth, follow-up timepoints were relatively limited, unable to characterise dynamic trajectories of depressive symptoms over time.

Future research should validate models in multicentre prospective cohorts, integrate biomarkers such as pro-inflammatory cytokines and KYN pathway metabolites, combine repeated measurement designs to depict symptom evolution processes, and ultimately evaluate real-world clinical utility through model-based, risk-stratified intervention studies.

## Conclusion

The machine learning-based models constructed in this study may help identify high-risk populations for postoperative depression in ovarian cancer. A clinical nomogram was additionally developed based on multivariable logistic regression, providing a visual, individualised scoring tool for bedside risk estimation. History of depression or anxiety, postoperative fatigue, education level, serum albumin, and operation time were identified as independent predictors. Pain, insomnia, inflammation–nutrition status, and previous mental health history represent key predictive factors, suggesting postoperative depression has multifactorial characteristics. Model-based risk stratification holds promise for providing more targeted interventions for high-risk patients. It should be emphasised that this model serves only as an auxiliary tool for clinical decision-making, with its clinical application value requiring further validation in prospective studies.

## Availability of Data and Materials

The data that support the findings of this study are not publicly available due to ethical and privacy restrictions involving patient information but are available from the corresponding author upon reasonable request and with appropriate institutional approval.
